# A conditional predictive *p*-value to compare a multinomial with an overdispersed multinomial in the analysis of T-cell populations

**DOI:** 10.1093/biostatistics/kxt039

**Published:** 2013-10-04

**Authors:** Qinglin Pei, Cindy L. Zuleger, Michael D. Macklin, Mark R. Albertini, Michael A. Newton

**Affiliations:** Department of Statistics, University of Wisconsin, Madison, WI 53706, USA; Department of Medicine and Carbone Cancer Center, University of Wisconsin, Madison, WI 53792, USA; Departments of Statistics and of Biostatistics and Medical Informatics, University of Wisconsin, Madison, WI 53706, USA

**Keywords:** Bayesian *p*-value, Dirichlet multinomial, Double overdispersion, Fisher's exact test, HPRT assay, Mass culture experiments, Molecular sequence data, T-cell receptor

## Abstract

Immunological experiments that record primary molecular sequences of T-cell receptors produce moderate to high-dimensional categorical data, some of which may be subject to extra-multinomial variation caused by technical constraints of cell-based assays. Motivated by such experiments in melanoma research, we develop a statistical procedure for testing the equality of two discrete populations, where one population delivers multinomial data and the other is subject to a specific form of overdispersion. The procedure computes a conditional-predictive *p*-value by splitting the data set into two, obtaining a predictive distribution for one piece given the other, and using the observed predictive ordinate to generate a *p*-value. The procedure has a simple interpretation, requires fewer modeling assumptions than would be required of a fully Bayesian analysis, and has reasonable operating characteristics as evidenced empirically and by asymptotic analysis.

## 1. Introduction

When testing the equality of two discrete populations, Fisher's exact test applies naturally to multinomial samples (e.g. Agresti, 1990, p. 62). It is widely used, easily developed, and readily interpreted, but it lacks robustness of validity when sources of variation create overdispersion relative to the multinomial. A more suitable model-based test is available in a case study from immunology that motivates the present work. One of the interesting features of the problem addressed concerns distributional shifts expected in the alternative hypothesis. If the biology is as suspected, then one population becomes a reduced-entropy version of the other. That is, probability masses in one population are concentrated on fewer support points when compared with the other population. But a similar concentration is a consequence of the overdispersion mechanisms governing data generation, even if the null hypothesis holds. Separating these effects to deliver refined statistical inference is the goal of the present work. Given the availability of powerful sequencing technology, we expect that data of this type will continue to be generated, and thus we are motivated to develop appropriate statistical methodology.

We address the testing problem by developing a conditional predictive *p*-value. Briefly, this involves splitting the data into two pieces. We condition on one piece to drive posterior inference for unknown parameters as well as predictive inference for the second piece of data; the predictive ordinate at the observed data acts as a test statistic. The approach is effective in the case study considered and exhibits good operating characteristics as determined through simulation and asymptotic analysis.

## 2. Biological context

Research on cancer immunotherapy aims to understand and enhance those components of the adaptive immune system that recognize and attack tumor cells. Recognition is through T cells, whose cell-surface receptors enable them to bind antigen, in the context of the appropriate antigen-presenting molecule, and initiate an immune response. The task of identifying T cells that are reactive to tumor-cell antigens is complicated by the tremendous diversity of the T-cell repertoire within the body. It is estimated that 10^12^ T cells constitute a human, with possibly 10^7^ distinct clonal types from among 10^18^ possibilities. This diversity is caused by combinatorial and stochastic processes by which T cells mature in the thymus, and it is manifested in a variation of DNA that encodes the *α* and *β* components of the T-cell receptor (e.g. Robins *and others*, 2009; Venturi *and others*, 2011). Of interest in the experiments summarized below are the J (joining) and V (variable) regions of the complementarity determining region 3 (CDR3) of the T-cell receptor *β* chain, which exhibits hypervariability and contributes to the antigen specificity of the T cell.

A T cell proliferates after it has been activated by recognition of its cognate antigen, and it produces a clone of descendants that share its specific cell-surface receptor. Thus, a promising approach to narrow the search for tumor-reactive T cells is to select from a patient's blood sample those T cells that have undergone post-activation cell proliferation. Assays have been developed based on somatically acquired mutations in the hypoxanthine-guanine phosphoribosyltransferase (*HPRT*) gene (Albertini *and others*, 2001, 2008). Briefly, cells that have incurred a loss-of-function mutation in *HPRT* are resistant to the effects of the nucleoside analog 6-thioguanine (6-TG), which otherwise is lethal to the cell. The frequency of *HPRT* mutant (MT) T cells is affected by various factors, is in the range 10^−6^ to 10^−5^ per mononuclear cell, and is readily estimated from a patient's sample via MT frequency analysis (Albertini, 2001). The rationale for *HPRT*-based selection is that, when compared with quiescent T cells, proliferating T cells are at an increased risk for having somatic gene mutations. The *HPRT* MT T cells therefore should be enriched for proliferating cells compared with wild-type (WT) T cells that have not been selected with 6-TG. In the analysis of data from these selection experiments, an important baseline statistical test is of the null hypothesis that MT and WT cell populations are *not* different with respect to frequencies over possible cell-surface receptors. It is prudent to perform the test as a check on the efficacy of the approach.

Recently, our group sequenced T-cell receptor CDR3's from cells in several compartments from six melanoma patients (Zuleger *and others*, 2011). We reconsider here data from the blood compartment in order to carefully develop a test of equality of discrete MT and WT populations. In the Zuleger *and others* (2011) data, counts of various types of CDR3's would have had multinomial distributions if the receptor sequences were obtained from random samples of MT and WT cells. However, the mass culture (MC) conditions used to propagate cells *in vitro* induced additional variability in the MT-type counts. The initial sample of cells from each donor was distorted by two related factors in culture. First, 6-TG treatment created a population bottleneck by killing all but the small fraction of input cells that carried an *HPRT* mutation. Second, cells were grown back to sufficient number to enable receptor sequencing. There was the possibility that different clones had different *in vitro* growth rates, and if so the type frequencies at the sampling/sequencing time would have differed from those of interest immediately after 6-TG treatment.

Receptor sequence data were obtained by two protocols; the MC method, indicated above, created extra-multinomial variation in MT counts. A second protocol was applied to a subset of sampled material; the single-cell (SC)-derived isolates produced less sequence data but were not subject to the overdispersion phenomenon. Whereas MC consists of culturing T cells from the blood as a bulk population, SC involves plating blood cells at limiting dilutions so that the derived monoclonal cultures can be sequenced and utilized in functional assays. Further details are provided in Section S2 of supplementary material available at *Biostatistics* online.

## 3. Data structure, sampling model, and prior

### 3.1 Data

Receptor sequencing was performed separately on material from different tissue samples. With *i* indexing the tissue sample and *t* indexing the type of the CDR3 receptor, the primary observable data are counts $\{ X_{i,t}^{\mathrm {mc}}, Y_{i,t}^{\rm mc}, X_{i,t}^{\rm sc}, Y_{i,t}^{\rm sc} \}$, which for sample *i* and CDR3 type *t* count the number of sequences of that type in that sample by one of two methods (SC and MC) and in one of two cell types (WT and MT).

\begin{align*} &X_{i,t}^{\mathrm{mc}} \longleftrightarrow \mbox{WT cells; MC,} \\ &Y_{i,t}^{\rm mc} \longleftrightarrow \mbox{MT cells; MC,} \\ &X_{i,t}^{\rm sc} \longleftrightarrow \mbox{WT cells; SC-derived isolates,} \\ &Y_{i,t}^{\rm sc} \longleftrightarrow \mbox{MT cells; SC-derived isolates}. \end{align*}

At the finest scale, *t* records the specific CDR3 amino-acid sequence; several thousand distinct *t*'s were observed in Zuleger *and others* (2011). To increase expected type counts, we performed analysis at coarser scales, in which *t* records any one of many distinct CDR3 sequences sharing a specific structural property. The J-region of the CDR3 assumed one of 13 possible structures; the V-region assumed one of 48 possibilities. Owing to experimental exigencies, a possibly different amount of sequencing was performed in each sample *i* on study. With reference to the above notation, the total numbers sequenced were

\begin{align*} m_i^{\mathrm{mc}} &= \sum_t X_{i,t}^{\rm mc}, \quad n_i^{\rm mc} = \sum_t Y_{i,t}^{\rm mc}, \\ m_i^{\rm sc} &= \sum_t X_{i,t}^{\rm sc}, \quad n_i^{\rm sc} = \sum_t Y_{i,t}^{\rm sc} . \end{align*}

Tables 1 and 2 show MC and SC J-region counts from six melanoma patients. V-region data on these patients are in Tables S1 and S2 of supplementary material available at *Biostatistics* online.

**Table 1.** Data: frequencies of J-region types, melanoma patients, blood, MC method

	A		B		C		D		E		F	
Subject J \Cells	WT	MT	WT	MT	WT	MT	WT	MT	WT	MT	WT	MT
1-1	15	49	9	1	9	71	14	0	7	11	16	0
1-2	8	0	4	1	9	1	13	2	10	2	6	2
1-3	0	1	6	3	1	2	5	0	5	1	2	0
1-4	1	0	1	0	1	0	2	18	1	0	2	6
1-5	5	2	3	0	5	0	8	0	7	7	12	0
1-6	0	1	1	0	4	2	3	0	9	0	1	3
2-1	12	5	12	70	14	0	12	10	14	14	13	22
2-2	13	4	8	10	5	5	5	1	10	2	2	55
2-3	12	7	9	0	10	0	8	56	7	14	6	0
2-4	1	0	0	0	2	0	0	0	1	0	1	0
2-5	3	7	10	0	16	0	2	1	4	0	5	0
2-6	1	0	1	0	2	5	1	0	2	0	0	0
2-7	18	5	21	1	13	6	19	7	20	31	12	19
Totals	89	81	85	86	91	92	92	95	97	82	78	107

WT and MT data arise from the same underlying discrete population on the null hypothesis, but MT data are subject to extra-multinomial variation.

**Table 2.** Data: frequencies of J-region types, melanoma patients, blood, SC-derived isolates method

	A		B		C		D		E		F	
Subject J \Cells	WT	MT	WT	MT	WT	MT	WT	MT	WT	MT	WT	MT
1-1	3	6	3	3	3	3	6	9	11	7	1	4
1-2	2	3	0	9	1	5	1	8	4	7	2	6
1-3	1	1	0	0	0	0	0	2	0	2	1	1
1-4	0	0	0	0	1	6	2	1	4	1	1	0
1-5	0	3	0	1	0	2	2	0	3	6	4	1
1-6	1	1	1	3	0	3	1	4	1	7	2	3
2-1	3	12	1	2	3	4	2	7	5	5	4	18
2-2	1	6	0	13	5	4	1	1	4	2	4	11
2-3	2	3	2	2	3	8	1	6	4	9	3	13
2-4	1	0	0	0	0	0	0	0	1	1	1	0
2-5	3	5	2	1	3	10	1	4	2	7	3	7
2-6	1	1	0	0	0	0	0	2	0	1	2	0
2-7	5	5	2	5	0	10	0	19	10	10	4	5
Totals	23	46	11	39	19	55	17	63	49	65	32	69

As in Table 1, but we are justified to treat these SC-derived data as multinomially distributed.

### 3.2 Sampling model

The primary inference task was to compare MT and WT populations using the count data. To this end, let $\theta = \{ \theta _t : t \in \mathcal {T}\}$ denote a vector recording the underlying proportion of each CDR3 type in the population being sampled. We worked on the hypothesis that this population was the same for WT and MT cells, and we sought to quantify evidence against this null. The issue of whether or not different individuals could be assumed to share this common *θ* was important, and one certainly expects fluctuation at some level; however, the magnitude of such fluctuations was small. For example, a Fisher test of common *θ* among subjects based upon the WT-SC data, which should not be affected by multinomial overdispersion, showed no substantial violations of the common-*θ* assumption (see Section S3 of supplementary material available at *Biostatistics* online). Thus, we retained a single *θ* vector in subsequent computations.

Considering the sampling and measurement process, and assuming the null hypothesis that MT and WT populations are equivalent, we expected the following:

\begin{align*} X^{\mathrm{mc}}_{i} &= \{ X^{\rm mc}_{i,t} : t \in \mathcal{T} \} \sim \mbox{Multinomial } (m_{i}^{\rm mc}, \theta), \\ X^{\rm sc}_{i} &= \{X^{\rm sc}_{i,t} : t \in \mathcal{T}\} \sim \mbox{Multinomial } (m_{i}^{\rm sc}, \theta), \\ Y^{\rm sc}_i &= \{Y^{\rm sc}_{i,t} : t \in \mathcal{T}\} \sim \mbox{Multinomial } (n_{i}^{\rm sc}, \theta). \end{align*}

We did not expect the counts $Y^{\rm mc}_i$ (those from MT cells grown in MC) to follow this sampling model, since *in vitro* effects induced extra-multinomial variation. The practical consequence was that $Y^{\mathrm {mc}}_{i,t}$ was very high for perhaps one or just a few sequence types *t* (e.g. J-region type 1-1 for patients A and C, Table 1). Interestingly, the research hypothesis is that the MT population is enriched for expanding (thus large) clones, and so we expected such clustering of sequence data if the immune system was actively responding to the melanoma. A goal of the statistical inference was to assess evidence against the null hypothesis while accommodating experimental factors that induced non-null-like clustering even in the absence of differences between the MT and WT populations.

A bottleneck was created *in vitro* since, on the average, a small fraction $f_i$ of cells in sample *i* carried the necessary HPRT mutation to withstand the 6-TG treatment. This mutation frequency $f_i$ was estimated for each sample (see Table S3 of supplementary material available at *Biostatistics* online). The surviving population grew back in culture to yield sufficient numbers for receptor sequencing. Then a random sample of $n_{i}^{\mathrm {mc}}$ sequences was obtained from the cell population after it had undergone this *in vitro* growth, giving observable type counts $Y_{i}^{\mathrm {mc}} = \{ Y_{i,t}^{\rm mc}: t \in \mathcal {T}\}$.

To analyze the bottleneck/growth effects, consider latent random counts $Z_{i}^{\mathrm {mc}} = \{ Z_{i,t}^{\rm mc}: t \in \mathcal {T} \}$, which record how many cells of each type survived 6-TG treatment prior to expansion *in vitro*. These components should be approximately independent and Poisson-distributed, with

(3.1) \begin{equation*}\label{eq3.1} Z_{i,t}^{\rm mc} \sim \mbox{Poisson } (s_i \theta_t f_i), \end{equation*}

where $f_i$ is the mutation frequency, $\theta _t$ is the population fraction of cells of CDR3 type *t*, and $s_i$ is the number of cells subject to 6-TG treatment in sample *i* (see Table S3 of supplementary material available at *Biostatistics* online). If all clones grew *in vitro* at the same rate, then the observable MT counts $Y_{i}^{\mathrm {mc}}$ would be multinomially distributed, with rates proportional to each $Z_{i,t}^{\mathrm {mc}}$. Experimentally, it is known that growth rates can vary; we allowed the variation in each rate to depend on the latent $Z_{i}^{\mathrm {mc}}$ and a single overdispersion parameter $\phi >0$, through a Dirichlet-multinomial model. Specifically

(3.2) \begin{equation*}\label{eq3.2} P (Y_{i}^{\mathrm{mc}} = y | Z_{i}^{\rm mc} = z) = \frac{n^{\rm mc}_i !\, \Gamma (\phi \sum_t z_t)}{\Gamma(n^{\rm mc}_i + \phi \sum_t z_t)} \prod_t \left\{\frac{\Gamma(\phi z_t + y_t)}{y_t !\, \Gamma(\phi z_t)}\right\}, \end{equation*}

for $y = \{ y_t : t \in \mathcal {T}\}$ counts summing to $n^{\mathrm {mc}}_i$ and for possible latent counts $z=\{z_t: t \in \mathcal {T}\}$. The Dirichlet-multinomial model has been used extensively for categorical data (e.g. Petkau and Sitter, 1989; Johnson *and others*, 1997, p. 80; Dunson and Tindall, 2000). It is a convenient model to accommodate extra-multinomial variation. Letting $\mu _t = z_t/\sum _s z_s$, it follows from (3.2) that

(3.3) \begin{equation*}\label{eq3.3} \begin{split} {\mathrm{E}} (Y_{i,t}^{\rm mc}|Z_{i}^{\rm mc} = z) &= n_i^{\rm mc} \mu_t, \\ {\rm var} (Y_{i,t}^{\rm mc}|Z_{i}^{\rm mc} = z) &= n_i^{\rm mc} \mu_t (1-\mu_t) \left(1 + \frac{n_i^{\rm mc} - 1 }{ \phi \sum_s z_s }\right). \end{split} \end{equation*}

The Dirichlet-multinomial model was also supported by subject-matter considerations. Suppose that type *t* is present in proportion $u_{i,t}$, say, in the population of cells from tissue sample *i* after several weeks expanding *in vitro*, where the latent vector of proportions $u_i=\{u_{i,t} : t \in \mathcal {T}\}$ has a Dirichlet distribution with parameters $\{ \phi z_t : t \in \mathcal {T} \}$. The random sampling of sequences after *in vitro* growth then gives a multinomial distribution for counts, conditionally upon $u_i$; the marginal Dirichlet-multinomial follows by integration. Furthermore, the Dirichlet model for cell population at sampling time was supported by models of species abundance, since a Gamma$(\phi z_t, 1)$-distributed population size for each clone would lead to the named Dirichlet distribution on vectors $u_i$ (Dennis and Patil, 1984). In allowing variable growth rates by this mechanism, we have assumed that the contribution to the sampled population from each cell that survives 6-TG selection has a Gamma(*ϕ*, 1) distribution.

A useful aspect of the Dirichlet-multinomial model is that it carries over to any collapsing of the categories. Specifically, let $\check {\mathcal {T}}$ be a set of collapsed categories $\check t = \{t \in \mathcal {T}: t\ \hbox { has specific property}\}$. Correspondingly, there are collapsed observed counts $\{\check Y^{\mathrm {mc}}_{i,t}\}$ and latent counts $\{ \check Z^{\mathrm {mc}}_{i,t} \}$ from the experimental data. It is a property of the Dirichlet multinomial that the distribution of $ \check Y^{\mathrm {mc}}_i$, given $ \check Z^{\mathrm {mc}}_i $, has the same form as in (3.2), but with *check*s everywhere instead! We say the sampling model for *Y* is *doubly* overdispersed, since there is extra-multinomial variation (caused by variable *in vitro* growth rates) even conditionally on the *Z* counts, and these counts are also unknown. When the surviving cell count $\sum _t Z_i^{\mathrm {mc}}$ is expected to be small, the variance of observed counts $Y_{i,t}^{\mathrm {mc}}$ is inflated both conditionally (through the variance inflation factor in (3.3)) and, marginally, through extra variation in $\mu _t$.

### 3.3 Prior

The sampling model from Section 3.2 involves unknown proportions $\theta = \{ \theta _t \}$ and an *in vitro*-growth parameter $\phi >0$. Numerical experiments (not shown) indicated that the posterior distribution of these parameters was not particularly sensitive to the prior setting. We report output in the case of a flat Dirichlet$(1,1, \ldots , 1)$ prior for *θ* and, independently, an improper flat prior for *ϕ*.

## 4. Conditional predictive *p*-value

We combine WT counts into a vector $X = \{ X_t \} $ over types *t* by adding contributions from $X_{i}^{\mathrm {mc}}$ and $X_{i}^{\mathrm {sc}}$ across all tissue samples. That is, $X_t = \sum _{i}(X_{i,t}^{\mathrm {mc}}+X_{i,t}^{\rm sc})$. Since we have combined multinomial counts governed by a common probability vector, the summary counts retain the multinomial form. Further, we let *Y* record the MC MT data $Y_{i}^{\mathrm {mc}}$ from all tissue samples (these are subject to overdispersion) as well as any available SC-derived counts $Y_{i}^{\mathrm {sc}}$. Here, we do not collapse by counting contributions over *i*, since such summarized counts would entail undue information loss; instead *Y* is a collection of vectors.

To emphasize notational distinctions, *X* and *Y* refer to random elements in our actual experiment, taking possible values *x* and *y*. As is sometimes done in *p*-value discussions, we introduce a separate notation for data obtained from a hypothetical repeat of the experiment: let $X^{\mathrm {rep}}$ denote a hypothetical repeated draw of the random vector *X*. Having observed $X=x$ and $Y=y$, the proposed *p*-value is

(4.1) \begin{equation*}\label{eq4.1} p_{\mathrm{cp}}(x,y)= P\{ p(X^{\rm rep}|y) \leq p(x|y) | Y=y \}, \end{equation*}

where $p(x|y)$ is a posterior predictive distribution for WT counts given observed MT counts:

(4.2) \begin{align} p(x|y) &= P(X=x|Y=y) \nonumber \\ &= \int P(X=x|\theta, Y=y) p(\theta|Y=y)\,{\mathrm{d}}\theta \nonumber \\ &= \int P(X=x|\theta) p(\theta|Y=y) \,{\rm d}\theta . \label{eq4.2} \end{align}

Here, *θ* is the vector of unknown probabilities over types. Although *x*, the possible realization of WT counts, is used above, nowhere have we conditioned on the event $\{X=x\}$. Had we conditioned in (4.1) instead on all the data $\{X=x, Y=y\}$, then the *p*-value would be a posterior predictive *p*-value (Gelman *and others*, 1996). This object can be unduly conservative, and so we decided to condition on part of the data only, as in the conditional predictive *p*-value approach (Bayarri and Berger, 1999, 2000; Evans, 2000; Robins *and others*, 2000). There, one conditions on part of the data, *U* (in our case, the MT counts *Y*), and generates a *p*-value from the conditional distribution of a statistic *T* that is as independent from *U* as possible. Our particular choice to split data by MT and WT counts and to construct a test statistic from the conditional density is most similar to Evans’ cross-validatory surprise (Evans, 2000), though examples and properties for count data seem not to have been developed previously.

A further justification of the proposed *p*-value (4.1) is its structural similarity to Fisher's exact test *p*-value, which would be suitable in the absence of multinomial overdispersion. Following the notational conventions above, Fisher's *p*-value is

(4.3) \begin{equation*}\label{eq4.3} p_{\mathrm{Fisher}}(x,y) = P\{ p(X^{\rm rep}| s) \leq p(x|s) | S=s\}, \end{equation*}

where $S=X+Y$ holds the sufficient statistic vector for the null frequencies *θ*, and where $p(x|s)$ is a generalized hypergeometric mass function. In the absence of overdispersion, this *p*-value is exact in the frequentist sense of being dominated by the uniform distribution, but unaccounted sources of variation tend to deflate and invalidate $p_{\mathrm {Fisher}}$. Splitting the data into its natural components *X* and *Y* enables construction of a conditional *p*-value that is similarly reliant on a conditional mass function as a test statistic. Further, in conditioning on one of the data components, it is more sensible to condition on *Y*, as we propose, since *Y* contains information on the frequency parameters *θ* as well as the overdispersion parameter. The WT data *X* informs only *θ*, on the other hand conditioning on *X* instead of *Y* would make the computation more difficult and more sensitive to prior information.

The null sampling distribution of the conditional predictive *p*-value proposed in (4.1) is neither exactly uniformly distributed nor dominated by the uniform distribution. Since posterior simulation is used to average over unknown variables, this null distribution is difficult to determine. The proposed *p*-value is a valid frequentist *p*-value in the sense that it converges to a uniform distribution as sample sizes diverge (Section 6). A fully Bayesian test of homogeneity between MT and WT proportions provides an alternative approach to the inference problem. This could be pursued since Bayesian analysis is already used to generate conditional predictions. We take a different approach, partly because a full Bayesian development would require further non-null modeling assumptions. Gelman and Shalizi (2012) discuss this and related factors supporting the use of predictive *p*-values.

## 5. Posterior and predictive sampling

Calculating the conditional predictive *p*-value (4.1) involves sampling hypothetical WT data vectors $X^{\mathrm {rep}}$ from their conditional distribution, given the observed MT data structure $Y=y$. It also requires repeated evaluation of the predictive ordinate $p(X^{\mathrm {rep}}|y)$ as well as the predictive ordinate on the observed WT data $p(x|y)$. We achieve the first task by Markov chain Monte Carlo (MCMC) coupled with predictive simulation. Specifically, $X^{\mathrm {rep}}$ has a multinomial distribution conditional upon *θ*, which we readily sample after MCMC delivers a sample of *θ* vectors from the posterior $p(\theta |Y=y)$. The marginal posterior of *θ* is naturally sampled by working with the higher-dimensional posterior of $(Z,\theta ,\phi)$, where *Z* is the data structure recording all the latent MT counts immediately after 6-TG treatment and prior to *in vitro* growth (Section 3.2), and where *ϕ* is the overdispersion parameter related to variability in clonal growth rates. Briefly, each scan updated *Z*, *θ*, and *ϕ* in blocks according to a Metropolis–Hastings sampler (e.g., Robert and Casella, 1999, Chapter 6). The target posterior distribution was $p(z,\theta ,\phi |Y=y)$, that is, the distribution of unknowns given the MT count data. Details of the proposal distributions, algorithm structure, and output diagnostics, and code are presented in supplementary material available at *Biostatistics* online.

The second task in evaluating $p_{\mathrm {cp}}(x,y)$ is to compute the predictive ordinate $p(x^*|y)$, both for the realized WT data $x^*=x$ and the many realized conditional predictions $x^*=x^{\rm rep}$ (as in (4.2)). We eschew Monte Carlo approximations for this purpose and instead use a simple numerical approximation associated with treating $p(\theta |Y=y)$ as a Dirichlet distribution. Realized *θ* vectors from the MCMC sampling are summarized to give a method-of-moments approximation to the parameters of this Dirichlet. Then the predictive ordinate is the ordinate of a Dirichlet-multinomial distribution, as the integration is achieved analytically. Details are provided in supplementary material available at *Biostatistics* online.

**Fig. 1.**
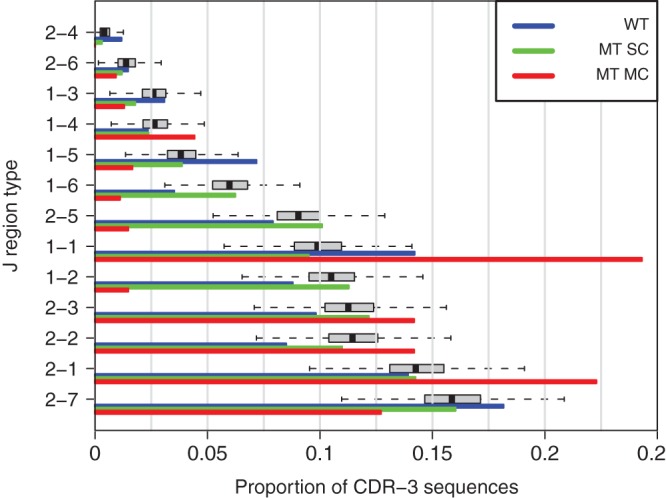
Summary statistics for J region frequencies from Tables 1 and 2. Colored bars show empirical frequencies of each J region type, from various data sources. Boxplots summarize posterior analysis of the underlying proportions conditional on the MT (not WT) data. Types are arranged from top to bottom by increasing value of the posterior median proportion. Note that the boxplots track the green bars better than the red bars, since the SC data are less variable than the MC data. The hypothesis test asks if the common (vector over types) mean of the MT data differs significantly from the mean of the WT cells (blue).

We applied the proposed computations to both J-region and V-region data from the six melanoma patients. Figure 1 summarizes features of $p(\theta |y)$ in comparison to empirical-type frequencies for the J-region data. (See Figure S1 of supplementary material available at *Biostatistics* online presents the V-region results.) Visually there is reasonably good agreement between MT and WT frequencies over different J-region types; substantial deviations (e.g. MT-MC compared with WT for types 1-1 and 2-1) reflect possible non-null behavior, though this must be gauged by intrinsic variations (e.g. see deviations between MT-MC and MT-SC). Balancing these factors, the Monte Carlo estimated conditional predictive *p*-value is $p_{\mathrm {cp}} = 0.046$, using 10^4^ saved draws from a well-mixed Markov chain (see Figure S2 of supplementary material available at *Biostatistics* online). In contrast to this borderline significance value, the V-region data give $p_{\mathrm {cp}} = 0 $ using the same simulation size. Evidently fluctuations as seen in the J-region data (Figure 1) are not particularly unusual on the null hypothesis of equal MT and WT frequencies. Significant deviations are evident in V-region data from patients.

The main point of the developed $p_{\mathrm {cp}}$ was to enable basic judgements about the MT/WT comparison in light of extra-multinomial variation. We note, for comparison, that direct application of Fisher's exact test would yield $p_{\rm Fisher}=0.00096$ (J) and $p_{\mathrm {Fisher}} < 2.2e-16$ (V), but this method is not reliable in the present context. For another comparison, we approximated the likelihood ratio statistic. Maximum likelihood estimation (MLE) is difficult in this hierarchical model. However, we approximated the MLEs using the posterior mean parameter settings from the developed MCMC sampler, separately for null and alternative hypotheses, and we used forward simulation to calculate likelihood components marginalizing the latent counts in *Z* (see Section S5 of supplementary material available at *Biostatistics* online). This gives $p_{\mathrm {LR}} = 0.04$ (J) and $p_{\mathrm {LR}} < 10^{-10}$ (V), assuming $\chi ^2$ asymptotics.

To check the reliability of the proposed $p_{\mathrm {cp}}$, we did a small simulation experiment in which system parameters were taken to be the posterior means from the J-region data and where sample sizes similarly matched the J-region data. MCMC sampling and predictive simulation were performed on each simulated data set. Figure S3 of supplementary material available at *Biostatistics* online illustrates the empirical distribution of 1000 null simulated $p_{\mathrm {cp}}$'s, and confirms that this estimated distribution is very close to uniform. To assess power, we also simulated the distribution of $p_{\mathrm {cp}}$ under the alternative, taking separate estimates of *θ* for MT and WT cells in order to match as closely as possible the experimental setting (see Section S5 of supplementary material available at *Biostatistics* online). We found a 0.67 and 0.84 probability for $p_{\mathrm {cp}} \leq 0.01$ and $p_{\mathrm {cp}} \leq 0.05$, respectively, in this case.

## 6. Asymptotic theory

Asymptotic uniformity of the proposed *p*-value can be proved using a curious fact about the large-sample distribution of the multinomial density of a multinomial sample. Fix $\theta =(\theta _1, \theta _2, \ldots , \theta _K)$ subject to $\theta _j>0$ and $\sum _j \theta _j=1$, and let $X^n=(X_1^n, X_2^n, \ldots , X_K^n)$ denote a multinomially distributed random vector on $n$ trials with probability vector *θ*. Recall the probability mass function

\[ p(x|\theta) = \frac{n!}{\prod_j x_j! } \prod_j \theta_j^{x_j}, \]

for suitable count vectors *x*.

**Lemma 6.1**

For the non-random sequence $c_n = (K-1) \log (2 \pi n) + \sum _{j=1}^K \log (\theta _j $*,
*

\[ -2 \log p(X^n|\theta) - c_n \longrightarrow_d \chi^2_{K-1}, \]

as $n\longrightarrow \infty $, where $\chi ^2_{K-1}$ is a $\chi ^2$ distributed random variable on $K-1$ degrees of freedom.

A proof is given in supplementary material available at *Biostatistics* online (Section S6). As MT sample sizes increase, the posterior $p(\theta |Y=y)$ converges to a point mass at the true *θ* vector, by standard large-sample Bayesian theory (e.g. Schervish, 1995, p. 428). Thus, the predictive ordinate $p(x|y)$ is indistinguishable from the ordinate of the true multinomial distribution $p(x|\theta)$. Taking logs and centering as in Lemma 6.1, the predictive *p*-value $p_{\rm cp}(X,Y)$, now considering inputs as random elements *X* and *Y*, is asymptotically equal in distribution to $h(V)$, where

(6.1) \begin{equation*}\label{eq6.1} h(v) = P (f(U) \leq f(V) | V=v), \end{equation*}

where *U* and *V* are independent and identically distributed $\chi ^2$ variables on $K-1$ degrees of freedom, and where $f$ is the density of this common distribution. The association is *V* is the limiting centered log mass $p(X|\theta)$ and *U* is the limiting centered log mass of $p(X^{\mathrm {rep}}|\theta)$. The uniformity of $h(V)$ is immediate from the probability integral transform. Note that MT data *Y* enter the game only to infer the parameter *θ*; otherwise, testing is left up to the WT data.

Our first attempt to obtain a useful test of the MT/WT difference used a different construction that exhibited unusual sampling properties. We imputed missing counts *Z* as in the proposed method, conditionally upon the MT data, but for each of these we calculated the Fisher test *p*-value that would be suitable in the absence of missing data. Then we averaged the resulting *p*-values. Computational experiments indicated that this *p*-value might be adjustable to provide a valid test; however, a detailed mathematical analysis uncovered a sampling defect associated with plugging in a consistent parameter estimate. The calculation is tangential to our main argument, but it was helpful in guiding us to a more useful construction, and so we include it in supplementary material available at *Biostatistics* online (Section S6).

## Supplementary material

Supplementary Material is available at http://biostatistics.oxfordjournals.org.

## Funding

The work was supported in part by the Office of Research and Development, Biomedical Laboratory Research and Development Service, Department of Veterans Affairs; grant P30 CA014520 from the National Cancer Institute; grant R21 HG006568 from the National Human Genome Research Institute; Anns Hope Foundation; the Gretchen and Andrew Dawes Melanoma Research Fund; the Jay Van Sloan Memorial from the Steve Leuthold Family; and the Tim Eagle Memorial. The contents do not represent the views of the Dept. of Veterans Affairs or the United States Government. C.L.Z. was supported by a postdoctoral fellowship by U.S. PHS Grant T32AR055893. Funding to pay the Open Access publication charges for this article was provided by University of Wisconsin fund which is called the Kellett Award.

## Supplementary Material

Supplementary Data
