# Supplementary material for: A conditional predictive p-value to compare a multinomial with an overdispersed multinomial in the analysis of T-cell populations
Source: Biostatistics. 2013 Oct 4;15(1):129–39. doi: 10.1093/biostatistics/kxt039 (PMC3862212; doi:10.1093/biostatistics/kxt039)
Supplement: Supplementary Data [file supp_15_1_129__index.html]

A conditional predictive p-value to compare a multinomial with an overdispersed multinomial in the analysis of T-cell populations — Supplementary Data 

# A conditional predictive *p*-value to compare a multinomial with an overdispersed multinomial in the analysis of T-cell populations

## Supplementary Data

Supplementary Data

**Files in this Supplementary Material:**

- Supplementary Data - Pdf file
