# Supplementary material for: A conditional predictive p-value to compare a multinomial with an overdispersed multinomial in the analysis of T-cell populations
Source: Biostatistics. 2013 Oct 4;15(1):129–39. doi: 10.1093/biostatistics/kxt039 (PMC3862212; doi:10.1093/biostatistics/kxt039)
Supplement: Supplementary Data [file supp_kxt039_kxt039supp_data.pdf]

Supplementary Material

**Main paper:**	A conditional predictive p-value to compare a multinomial with an overdispersed multinomial in the analysis of T-cell populations
**Authors:**	Pei *et al.*
**Date:**	September 20, 2013

This supplement is organized into sections that refer back to the relevant section in the main paper. For example, the section S6 below refers to Section 6 in the main paper (Asymptotic Theory) and presents details of issues raised in that section. Reported figures and tables, both in the main paper and supplement, can be reconstructed using `R`'s `demo` facility and the accompanying `R` package, `dod` (short for *double overdispersion model*). The available `demo`'s are:

```
demo(datatables)              create all data for tables
demo(figure1)                 create Figure 1 (fig1.pdf)
demo(figureS1)                create Figure S1 (figS1.pdf)
demo(figureS2)                create Figure S2 (figS2.pdf)
demo(figureS3)                create Figure S3 (figS3.pdf)
demo(lrstatJ)                 approximate likelihood ratio statistic; J region
demo(lrstatV)                 approximate likelihood ratio statistic; V region
demo(power)                   simulate non-null p-value distribution
```

Computations were done with `R` version 2.15.1 (R Development Core Team 2011). To simplify the package operation, results of the three heaviest computations (MCMC sampling for J and V, and the simulation) were precomputed and stored as data objects in a list called `precooked`, though the precise code to obtain the results is also provided in example scripts within `dod`.

S2: Study design and cell-culture protocols

Study design

There was no disease-related or data-related selection that was used for the samples analyzed. For the purposes of simplifying the model development, we chose to focus in the present manuscript on one highly relevant class of samples, namely blood samples from melanoma patients. All of the patients from whom sequence data were collected were analyzed here. The melanoma patients in this study were seen at the William S. Middleton Memorial Veterans Hospital or at the University of Wisconsin Hospital and Clinics between December 2004 and July 2006 and had biopsy-confirmed diagnoses of melanoma that was either American Joint Committee on Cancer (AJCC) Stage III or IV. Melanoma patients provided blood and/or tumor samples. Blood samples were also obtained from normal control blood donors at the William S. Middleton Memorial Veterans Hospital or at the University of Wisconsin Hospital and Clinics. The study was approved by the Health Sciences Institutional Review Board that serves both the William S. Middleton Memorial Veterans Hospital and the University of Wisconsin-Madison. Written informed consent was obtained from all participants. Data from control subjects were not reanalyzed in the present manuscript; neither were data from patient tumor samples, but these were reported in the original study paper, Zuleger et al. 2011.

Mass culture (MC) and single-cell derived isolates (SC)

Single-cell derived T-cell isolates provide a means by which to associate individual CDR3 to functional attributes of a particular T cell. This provides valuable insight in terms of T-cell specificity, affinity, lineage, and maturation status. Further, additional sequence analyses with single-cell derived isolates link gene expression to a particular T cell. As example, the TCR is composed of two chains which both contribute to the T-cell's specificity. Both chains can be sequenced from a single-cell derived T-cell isolate giving a more complete description of the TCR. However, T-cell cloning is not a trivial undertaking. We thus investigated the mass culture system, which is faster, cheaper, and less time consuming than working with T-cell isolates. Mass culture also permits functional T-cell analyses while taking advantage of cell-to-cell interactions and a somewhat more physiological system (i.e., cells do not exist in isolation in vivo). Although the mass culture does not permit the association of particular CDR3's with function, the trade-off is high-throughput analysis. Further, mass culture selection can be applied to blood, to lymph nodes, and to other tissues of interest for which limited cell numbers may preclude T-cell cloning.

The MC consists of culturing T cells from the blood as a bulk population in the presence (MT) or absence (WT) of 6-TG until sufficient cell numbers are available for sequencing. Typically the WT culture is initiated with 1×10^5 total cells, whereas the MT is initiated with at least 5×10^6 total cells. The SC are obtained by plating blood cells in 96-well round-bottom plates in the presence (MT) or absence (WT) of 6-TG. WT SC are plated at limiting dilution cell concentrations (i.e., 1-, 2-, or 5-cells per well). MT SC are plated at 10,000 cells per well. Plates are cultured for 14 days at which time expanding T cells are identified and further expanded prior to sequencing. These protocols have been described in detail (Albertini *et al.* 2008, and O'Neill *et al.* 1987).

S3: Data structure and sampling model

Supplementary Data

Tables 1 and 2 in the main paper summarize J-region type frequencies for MT and WT CDR3 sequence data from six melanoma patients. Tables S1 and S2 present comparable frequencies when sequence data is organized by V-region type. (Patients A-F here correspond to patients P-13, P-9, P-5, P-3, P-2, and P-1 in Zuleger *et al.*, 2011.)

In the case examined MC and SC data were measured for both MT and WT cells from all subjects. The methodology allows imbalance in these data sources and the possibility that some source (e.g. SC) is not measured on every sample.

Table S3 reports the cell characteristics for all the samples reconsidered here.

Common θ diagnostic

The sampling model (Section 2, main paper) ignores variation among patients in the underlying proportion vector θ. There is some support for this simplifying assumption, as evidenced by a look at the single-cell derived isolates (SC) data from untreated samples (WT). These data should be least affected by extra-multinomial sources of variation. The J-region counts for this case are

```
    A B C D  E F
2-2 1 0 5 1  4 4
1-1 3 3 3 6 11 1
2-3 2 2 3 1  4 3
2-7 5 2 0 0 10 4
```

```
1-2 2 0 1 1  4 2
2-1 3 1 3 2  5 4
1-5 0 0 0 2  3 4
1-4 0 0 1 2  4 1
1-6 1 1 0 1  1 2
2-5 3 2 3 1  2 3
1-3 1 0 0 0  0 1
2-6 1 0 0 0  0 2
2-4 1 0 0 0  1 1
```

The following `R` script using `dod` does the Fisher test for equality of the column proportion vectors:

```
data(patients)
J <- patients$Jcounts$"WT-SC"
checkJ <- fisher.test( J, simulate.p.value = TRUE )
```

which evaluates to $p = 0.48$. The calculation for V-region data gives $p = 0.71$. Thus we ignore patient effects at J and V levels of resolution.

S5: Sampling methods

Posterior sampling

Adopting standard notational simplifications and the double over dispersion model from the main paper, the target posterior distribution is

$$p(z, \theta, \phi | y)$$

where z holds a matrix (types by patients) of latent counts just prior to *in vitro* growth (and just after 6-TG treatment) for the MT cells, θ is the vector (over types) of type frequencies, ϕ is an overdispersion parameter (Gamma shape parameter), and y is the set of all MT data being conditioned upon for this phase of the analysis. We implement a standard block-updating sampler, cycling through

1. $p(z|\text{else})$
2. $p(\theta|\text{else})$
3. $p(\phi|\text{else})$.

The simplest of these is the θ update, since under the flat Dirichlet prior this full conditional distribution is again Dirichlet, involving total counts of both observed MT-SC data and imputed z's underneath the MT-MC data.

The univarite ϕ parameter is updated with a random-walk Metropolis sampler in a small uniform window around the current value. Numerical experiments lead us to settle on window size for production runs.

For the latent matrix z, we sample one patient's count vector to be modified, and we simply propose to modify each type's latent count to be a draw from that variable's marginal Poisson distribution (given θ). Then we adjust this proposal according to the Dirichlet-Multinomial likelihood

component to retain detailed balance against $p(z|\text{else})$. We experimented with modifying fewer than all type counts at once, but found adequate mixing with the stated proposal.

Reported posterior summaries come from 10000 saved states subsampled every 500 states after substantial burn in. MCMC diagnostics supported this design (Figure S2). In the `dod` package, see `help(MCMC)` for specific implementation.

In R version 2.15.1, and on a 2.53GHz Intel Xeon CPU, the J-region MCMC run used 43.8 CPU minutes and the V-region MCMC run used 70.4 CPU minutes (5 million post-burn-in sweeps each; *user time* from `proc.time`).

Predictive sampling

The second step in computing the conditional predictive p-value involves the evaluation of the predictive mass function

$$p(x^*|y) = \int p(x^*|\theta)\, p(\theta|y)\, d\theta \tag{1}$$

for various vector inputs x^* (both the observed WT data as well as simulated versions). Considering the relative complexity of the double-overdispersion model for y, it is apparent that $p(\theta|y)$ has no simple analytic form, in spite of the fact that our analysis allows us to sample it via MCMC. A computationally intensive and numerically unstable approach to evaluate 1 is to replace the integral with a Monte Carlo approximation using the MCMC output; we could do this both for x^* equal the observed WT data and for the numerous predictive simulations. Like the instability observed with importance sampling, there is instability by this approach; our experiments showed that one or just a very few of the sampled θ values would carry substantial weight $p(x^*|\theta)$ for any given x^*. Instead, we adopted the following approximation. We assumed that $p(\theta|y)$ could be approximated by some Dirichlet distribution. We used the posterior samples (obtained by MCMC) to approximate the parameters of this Dirichlet (using the method of moments). Then we took advantage of the fact that the integral in 1 has a closed form when $p(\theta|y)$ is Dirichlet and $p(x^*|\theta)$ is Multinomial. Indeed, it is precisely (and coincidentally) the the Dirichlet-Multinomial model analogous to the model used in the main paper (equation 3.2). The function `cpp` in the package `dod` gives the specific implementation:

```
cpp <-
function(fit)
 {
  ## estimate p(x|y) with a Dirichlet
  ## multinomial fitted using sampled theta's
  ##
  ## fit    result of running MCMC in double-overdispersion model

  ## WTcounts vector holding experimental CDR3 sequence counts, Wild Type cells
  wt <- fit$counts[[1]] + fit$counts[[2]]  ## add SC and MC WT counts, per subject
  WTcounts <- apply(wt,1,sum)  ## sum over subjects

  theta.mean <- apply( fit$thetasave, 2, mean )
  theta.v <- var( fit$thetasave )
  dirtot <- median(   theta.mean*(1-theta.mean)/diag(theta.v) - 1 )
```

```
# next compute the conditional p-value by simulating Dirichlet multinomial
# WT counts

xstar <- apply( fit$thetasave, 1, rmultinom, n=1, size=sum(WTcounts) )

# predicted data density
logpred.star <- lgamma( xstar + dirtot*theta.mean ) - lgamma( xstar+1 )

# observed data density
logpred.x <- lgamma( WTcounts + dirtot*theta.mean ) - lgamma( WTcounts + 1 )

logpred.ratio <- apply( logpred.star - logpred.x , 2, sum )

pval <- mean( logpred.ratio <= 0 )

return(pval)
}
```

Approximate likelihood ratio statistic

For comparative purposes, we sought to evaluate the likelihood ratio test for the same problem. This is complicated by the fact that maximum likelihood estimation is difficult owing to the hierarchical model structure. To approximate MLE's, we used posterior mean estimates computed using the developed MCMC code. For the null case, we had to modify the MCMC code so that all MT and WT data were used to compute the estimated θ vector (in contrast to just the MT data which were used for p_{cp}). Since the WT data are multinomial this is a simple modification (*method=2* within the `MCMC` function in `dod`). Thus we approximated $\hat{\theta}^0$ (MLE under null) and $\hat{\theta}^{\mathrm{MT}}$ and $\hat{\theta}^{\mathrm{WT}}$ (MLEs under alternative), as well as the MLEs $\hat{\phi}^0$ and $\hat{\phi}^1$ of the overdispersion parameter (under the null and alternative).

With x and y denoting the observed WT and MT data sets, the LR statistic is

$$-2\left\{\log p(x,y|\hat{\theta}^0,\hat{\phi}^0) - \log p(x,y|\hat{\theta}^{\mathrm{WT}},\hat{\theta}^{\mathrm{MT}},\hat{\phi}^1)\right\}.$$

In both terms the joint mass function factors, and $p(x|\theta)$ takes the multinomial form. The difficulty is in $p(y|\theta,\phi)$, which is an expectation, over latent Z matrices, of Dirichlet-Multinomial masses $p(y|Z,\phi)$, where Z is a matrix with Poisson-distributed entries. This we approximate by forward simulation of the Poisson matrices. Finally we obtain p-values by treating the LR statistic as chi-square distributed on $K-1$ degrees of freedom, as would be valid asymptotically. Code to evaluate the approximate LR test is in `demo(lrstatJ)` and `demo(lrstatV)`.

We observed higher Monte Carlo error in the LR p-value than in the conditional predictive p-value. For example, 20 repeated runs of `cpp(precooked$fitJ)` had mean 0.046 with standard deviation 0.002; while 20 repeats of `demo(lrstatJ)` had mean 0.038 with standard deviation 0.011. As we were fixing the MCMC sample, this extra variation came from the forward simulation component of the LR statistic calculation.

Simulation

To check the null sampling distribution of p_{cp} we simulated the double-overdispersion model and repeatedly computed its value via posterior and conditional predictive sampling. Figure S3 shows

results for one case where the parameters were as estimated for the J-region data. The function `simdod` in the package `dod` gives the specific implementation:

```
simdod <-
function(nsim,theta,phi, sizes, mcmc )
 {
  ###############################################################################
  ## simulate the double overdispersion model in Pei et al 2012 and compute CPP
  ## for each data set
  ##
  ## nsim     number of simulated data sets
  ## theta    latent frequency of proportions (vector over CDR3 types)
  ## phi      overdispersion parameter for in vitro growth
  ##
  ## sizes$n.wtsc vector (number of subjects) holding# WTSC sequences per subject
  ## sizes$n.wtmc vector (number of subjects) holding# WTMC sequences per subject
  ## sizes$n.mtsc   vector (number of subjects) holding #MTSC sequences per subject
  ## sizes$n.mtmc   vector (number of subjects) holding #MTMC sequences per subject
  ## sizes$nsurvive   vector (number of subjects) # surviving cells per subject
  ##                          ## for MT MC part
  ##
  ## mcmc$nsave       # stored states in MCMC
  ## mcmc$nskip       # skipped states in MCMC
  ## mcmc$initc       # burn-in
  ## mcmc$phi.init    # initial value
  ## mcmc$phi.delta   # MH windo size

  pval <- numeric(nsim)

  for( isim in 1:nsim )
   {
    # make WT data
    wtsc <- sapply( as.list(sizes$n.wtsc), rmultinom, n=1, prob=theta )
    wtmc <- sapply( as.list(sizes$n.wtmc), rmultinom, n=1, prob=theta )

    # make MT SC data
    mtsc <- sapply( as.list(sizes$n.mtsc), rmultinom, n=1, prob=theta )
    # make MT MC data by first getting latent Z's
    muz <- theta %o% sizes$nsurvive
    zstate <- apply(muz,2,rpois, n=nrow(muz) )
    # next make Dirichlet distributed prob vector
    uu <- apply(phi*zstate,2,rgamma,n=nrow(muz) )
    uprob <- t( t(uu)/apply(uu,2,sum) )
    # next make the conditionally multinomial observations
    uplist <- as.list( data.frame(uprob) )  ## columns entries of list
    mtmc <-mapply( function(x,y) rmultinom(n=1,size=x,prob=y), sizes$n.mtmc, uplist )
    simcounts <- list( 'WT-SC'=wtsc, 'WT-MC'=wtmc,
'MT-SC'=mtsc, 'MT-MC'=mtmc ) ## simulated data
```

```
    # run MCMC  to get posterior of theta given MT (only) data Y=y

    fit.sim <- MCMC( simcounts, sizes$nsurvive, mcmc=mcmc,  method=1,
print.iter=FALSE)

    ## get conditional predictive p-value

    pval[isim] <- cpp( fit.sim )

    print(isim)
   }
  return( pval )
 }
```

The null simulation described above was readily modified to enable an assessment of power of p_{cp}. Two distinct θ vectors were required: one governing the multinomial WT data and one governing the more complex MT data. We used the posterior mean of θ as the driving value for simulated MT data (recall, this posterior relies only upon the observed MT data); we used the observed WT proportions vector (MLE, under alternative) as the governing parameter for simulated WT data. As in the null simulation, we used sample sizes from the original study design. Thus the data generation matched as well as possible the estimated non-null state for J-region data in our six patients. For each simulated data set (1000), we computed the conditional predictive p-value (using 20000 post-burn in sweeps in each case) and kept track of the empirical p-value distribution. See `demo(power)`.

Table S1: Data: frequencies of V-region types, melanoma patients, blood, mass culture (MC) method. WT and MT data arise from the same underlying discrete population on the null hypothesis, but MT data are subject to extra-multinomial variation.

Subject	A		B		C		D		E		F	
V \Cells	WT	MT	WT	MT	WT	MT	WT	MT	WT	MT	WT	MT
10-1	1	0	0	0	0	0	0	0	0	4	0	0
10-2	1	0	0	0	0	0	0	0	0	0	0	0
10-3	0	0	1	0	3	0	2	13	1	7	0	3
11-1	0	0	0	0	0	0	1	0	1	0	1	0
11-2	2	0	6	0	3	2	1	0	0	0	2	0
11-3	2	0	0	3	0	0	0	0	1	0	0	0
12-3	1	9	1	0	3	1	1	0	3	0	5	0
12-4	4	0	0	0	1	0	4	0	1	0	2	0
12-5	1	5	0	0	1	0	0	0	1	0	0	0
13	0	0	1	0	2	0	0	0	2	0	1	0
14	0	0	1	0	1	0	0	0	3	0	0	0
15	1	1	1	0	4	2	0	0	2	0	0	0
18	1	0	1	0	1	7	1	0	0	0	1	0
19	1	0	4	0	5	0	1	0	4	20	5	0
2	10	49	5	0	13	23	5	0	10	0	2	0
20-1	4	0	5	2	6	0	11	8	1	28	9	3
24-1	2	0	1	0	0	0	0	0	0	0	1	1
25-1	1	0	2	0	0	0	1	0	1	1	0	0
27	8	0	1	0	4	7	7	0	5	4	4	1
28	28	0	3	0	6	0	11	0	26	12	10	1
29-1	6	0	7	68	3	0	8	8	3	0	6	0
30	0	0	3	0	1	0	0	0	0	0	2	0
3-1	2	0	2	2	1	0	4	0	4	1	0	0
3-2	0	0	0	0	0	0	0	0	0	0	0	0
4-1	1	0	4	0	3	0	0	0	0	0	0	16
4-2	0	2	0	0	2	0	1	12	1	0	1	61
4-3	0	0	0	0	0	0	1	0	1	0	0	0
5-1	0	4	2	1	3	0	4	0	1	0	5	0
5-4	0	0	2	0	0	0	0	0	0	0	0	0
5-5	1	0	1	0	0	2	0	0	3	0	1	0
5-6	0	0	1	0	2	0	2	5	0	0	0	0
5-8	0	0	0	0	0	0	0	0	0	0	0	0
6-1	1	2	1	0	2	1	2	0	5	1	3	0
6-2	0	0	2	0	1	0	3	41	2	0	2	0
6-3	0	0	0	0	0	0	0	0	0	0	0	0
6-4	0	0	1	0	0	0	1	0	0	0	0	0
6-5	3	0	4	0	2	0	9	0	4	0	6	0
6-6	0	2	4	0	9	35	2	1	2	0	2	0
6-8	0	0	0	0	0	0	0	0	0	0	0	0
7-2	0	0	4	0	1	11	2	4	1	0	4	0
7-3	0	0	3	1	1	0	3	0	1	0	0	14
7-4	0	0	0	0	0	0	0	0	0	0	0	0
7-6	0	2	0	0	0	0	1	1	1	0	1	0
7-7	0	0	0	0	0	0	0	0	0	0	0	0
7-8	3	0	1	0	0	0	1	0	0	0	1	7
7-9	2	2	5	0	5	1	1	0	4	3	1	0
9	2	3	5	9	2	0	1	2	2	1	0	0
9-1	0	0	0	0	0	0	0	0	0	0	0	0
totals	89	81	85	86	91	92	92	95	97	82	78	107

Table S2: Data: frequencies of V-region types, melanoma patients, blood, single-cell-derived isolates (SC) method. As in Table S1, but we are justified to treat these SC-derived data as multinomially distributed.

Subject	A		B		C		D		E		F	
V \Cells	WT	MT	WT	MT	WT	MT	WT	MT	WT	MT	WT	MT
10-1	0	0	0	0	0	0	0	0	0	1	0	0
10-2	1	0	0	0	0	0	0	0	0	0	0	0
10-3	0	3	0	0	0	2	0	6	0	1	0	0
11-1	0	0	0	0	0	0	0	0	1	0	0	1
11-2	1	4	0	1	1	2	2	3	0	0	0	3
11-3	0	0	0	0	0	0	0	0	0	0	0	0
12-3	3	1	0	0	1	2	1	1	2	0	1	2
12-4	0	1	0	1	1	1	1	1	2	3	1	0
12-5	0	0	0	0	0	0	0	0	0	0	0	0
13	0	1	0	0	0	0	0	0	0	1	0	1
14	0	0	1	2	0	2	0	2	1	5	2	2
15	0	0	0	0	0	2	1	0	1	2	1	0
18	0	0	0	0	0	0	1	0	2	2	1	0
19	1	4	0	2	0	2	2	5	1	3	0	0
2	1	2	3	0	1	9	1	1	5	3	0	0
20-1	2	2	2	8	2	8	0	8	5	7	2	12
24-1	0	1	0	0	0	0	0	0	3	2	0	2
25-1	0	3	0	0	0	0	1	0	1	0	0	0
27	0	1	0	4	2	0	2	2	3	2	2	0
28	0	0	2	0	0	1	1	2	4	6	4	3
29-1	1	0	0	0	2	2	0	1	1	1	0	0
30	2	2	0	0	1	1	0	2	0	1	1	0
3-1	1	2	0	1	1	4	0	2	2	0	1	0
3-2	0	0	0	0	0	1	0	0	0	0	0	0
4-1	0	0	0	4	1	2	0	0	1	2	0	1
4-2	0	1	0	0	0	0	1	2	0	0	1	1
4-3	0	0	0	0	2	1	1	0	1	3	0	1
5-1	3	3	2	2	3	1	2	6	5	7	5	3
5-4	0	0	0	0	0	0	0	0	0	0	1	0
5-5	0	0	0	0	0	0	0	0	0	0	0	0
5-6	0	0	0	0	0	1	0	0	0	0	0	0
5-8	1	0	0	0	0	1	0	0	0	0	0	0
6-1	2	3	0	2	0	1	0	2	0	1	0	0
6-2	0	0	1	0	0	0	0	3	0	2	0	0
6-3	0	0	0	0	0	3	0	2	1	0	0	0
6-4	0	0	0	0	0	0	0	0	0	0	0	0
6-5	0	3	0	0	0	1	0	3	2	4	2	2
6-6	0	0	0	0	0	0	0	0	0	0	0	0
6-8	0	0	0	0	0	1	0	0	0	0	0	0
7-2	0	0	0	0	0	0	0	0	1	0	0	4
7-3	1	0	0	0	0	0	0	3	1	2	2	11
7-4	0	2	0	0	0	0	0	2	1	1	0	0
7-6	0	0	0	0	0	0	0	0	0	0	1	1
7-7	1	0	0	0	0	1	0	0	0	0	1	0
7-8	1	2	0	0	0	3	0	0	0	0	0	17
7-9	1	1	0	0	0	0	0	0	0	0	1	1
9	0	4	0	12	1	0	0	4	2	3	0	0
9-1	0	0	0	0	0	0	0	0	0	0	2	1
totals	23	46	11	39	19	55	17	63	49	65	32	69

Table S3: Mutant frequencies and cell culture characteristics

melanoma patient (blood)	A	B	C	D	E	F
mutant frequency ($\times 10^{-6}$)	48.5	40.3	39.4	20.0	23.45	97.3
number of cells at time 0 ($\times 10^{6}$)	20	10	10	10	10	5

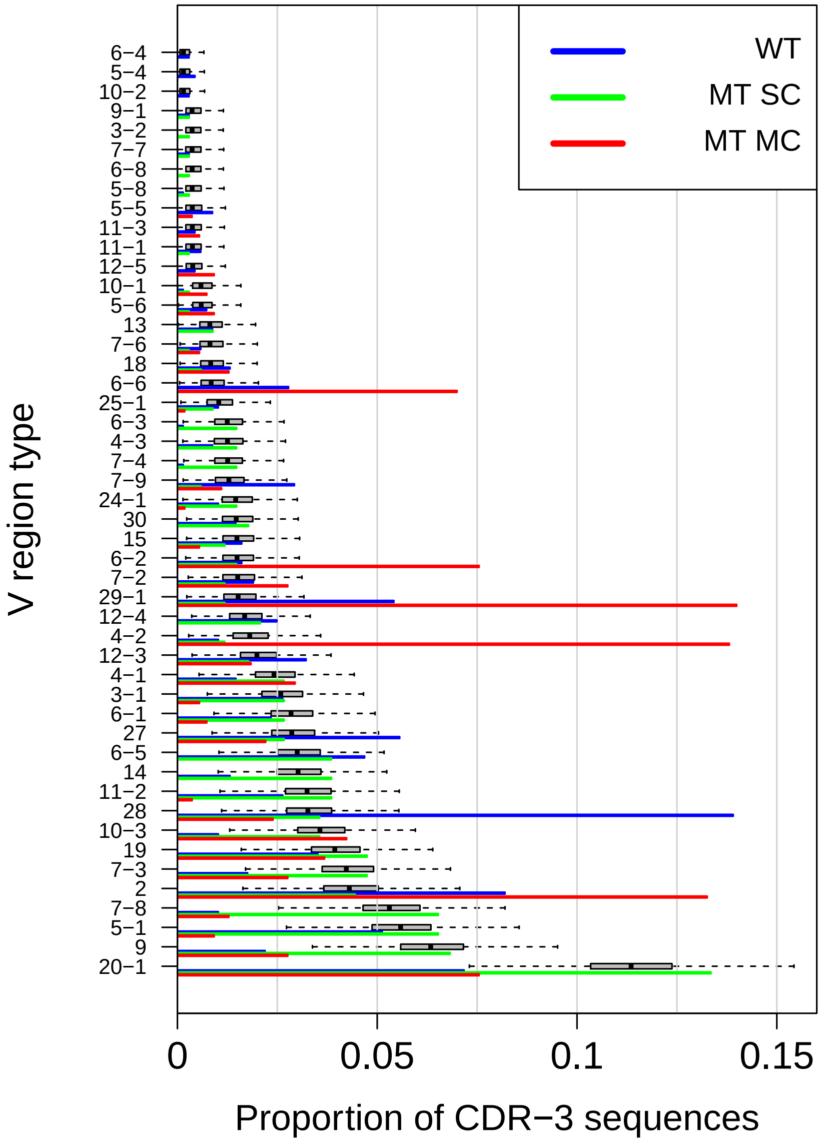

Figure S1: Summary statistics for V region frequencies from Tables S1, S2, similar to Table 1, which shows data for J regions. Colored bars show empirical frequencies of each V region type, from various data sources. Boxplots summarize posterior analysis of the underlying proportions conditional on the MT (not WT) data. Types are arranged from top to bottom by increasing value of the posterior median proportion. Note that the boxplots track the green bars better than the red bars, since the SC data are less variable than the MC data. The hypothesis test asks if the common (vector over types) mean of the MT data differs significantly from the mean of the WT cells (blue).

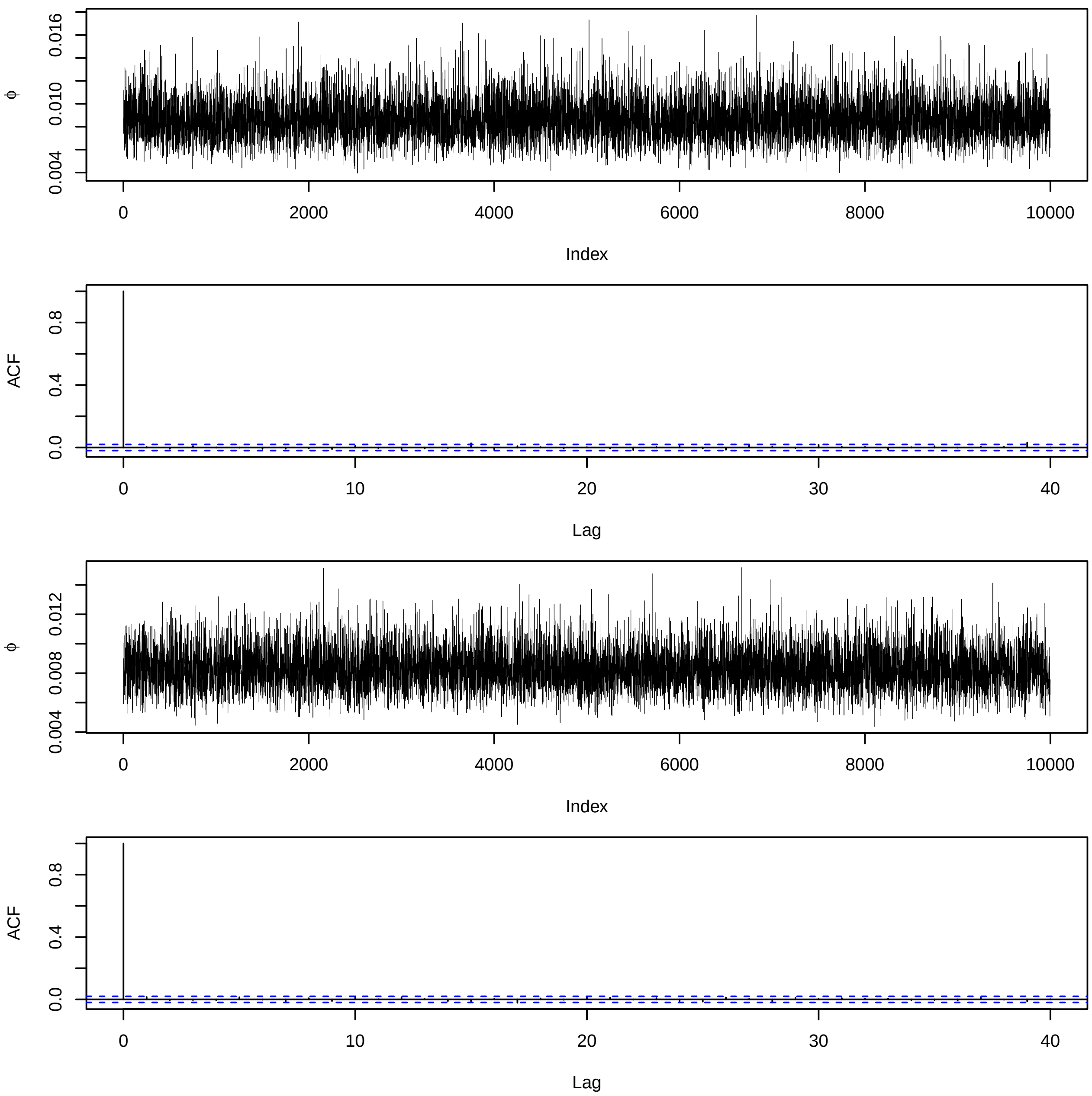

Figure S2: Basic trace plot MCMC diagnostics, for parameter ϕ under posteriors from J-region data (top panels) or V-region data (bottom panels). Trace plots and autocorrelation plots are consistent with excellent mixing of the MCMC. The sampler used $10,000$ saved scans from a chain of length $500 + 10,000 \times 500$.

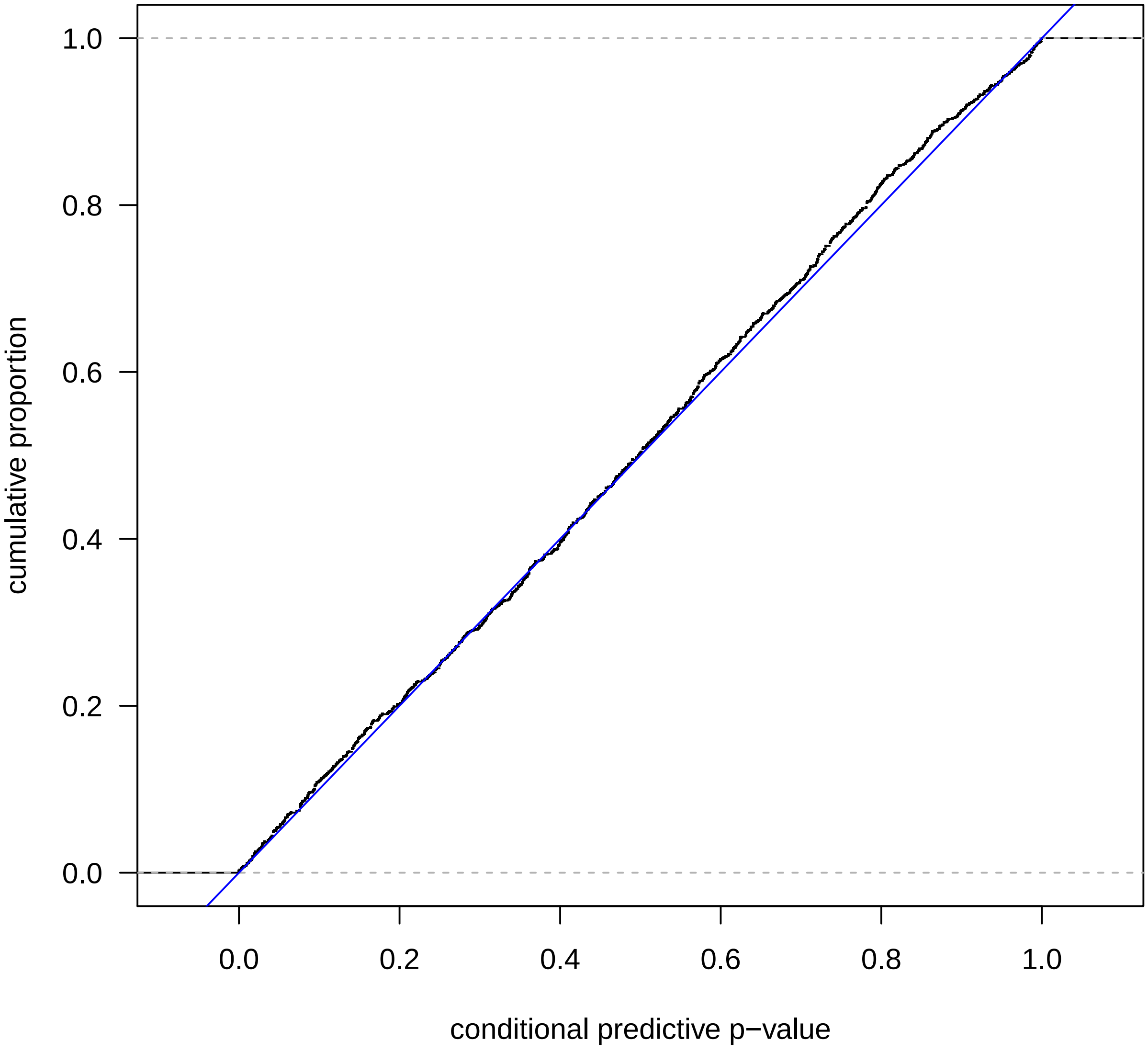

Figure S3: Simulated null distribution of conditional predictive p-value, with settings (i.e. sample sizes and parameters) matching those given and estimated for the J region patient data. 1000 data sets were simulated from the double overdispersion (dod) model (on the null). MCMC was used on each simulated data set to compute the conditional predictive p-value. 1000 states were saved after a short burn-in and subsampling every 20 complete scans. This and similar numerical experiments (not shown) demonstrate that the conditional predictive p-value has a frequency distribution that is approximately uniform

S6 Asymptotics

Proof of Lemma 6.1

From Stirling's approximation,

$$\log(m!) = \log(\sqrt{2\pi}) + \log(\sqrt{m}) + m\log(m) + o(1) \quad \text{as } m \to \infty.$$

Applying this to all factorials in the log mass function, we have

$$\log p(X^n|\theta) = (1-K)\log(\sqrt{2\pi n}) - \frac{1}{2}\sum_{j=1}^{K}\log\hat{\theta}_j + n\left\{\sum_{j=1}^{K}\hat{\theta}_j\log(\theta_j/\hat{\theta}_j)\right\} + E_n.$$

Here $\hat{\theta}_j = X_j^n/n$, the maximum likelihood estimate of θ_j, and E_n accumulates error terms and is converging in probability to 0 as $n \to \infty$ since all θ_j are strictly positive. The result follows immediately upon recognizing that minus 2 times the term preceding E_n is the likelihood ratio statistic, which is converging to the chi-squared limit according to standard theory (e.g., Bickel and Doksum, 1977, page 315.)

A different version of p_{cp}

Fisher's exact test would have enabled a comparison of WT and MT populations if the counts Z_i^{mc} were observable. Our first idea was to repeatedly recompute Fisher's p-value using these simulated latent counts, and then average the resulting p-values. Formally, we constructed a statistic according to the following argument.

Let $h(x,z)$ denote the generalized hypergeometric mass function in a $K \times 2$ contingency table with count vector x in one column and z in another. Specifically

$$h(x,z) = \frac{x.!z.!}{(x. + z.)}\prod_t \frac{(x_t+z_t)!}{x_t!\,z_t!}$$

where $\cdot$ indicates summation over the K distinct types t. Combine all WT counts into a vector $X = \{X_t\}$ over types t by adding contributions from X_i^{mc} and X_i^{sc} across all tissue samples. That is $X_t = \sum_i \left(X_{i,t}^{\text{mc}} + X_{i,t}^{\text{sc}}\right)$. Similarly combine MT data, but consider doing so with imputed counts Z_i^{mc} and with any available single-cell derived counts Y_i^{sc}, and call this vector Z; say $Z_t = \sum_i \left(Z_{i,t}^{\text{mc}} + Y_{i,t}^{\text{sc}}\right)$. In each case, we combine multinomial counts that have a common probability vector, and so we retain the multinomial form. Finally, let Y record the mass-culture MT data Y_i^{mc} from all tissue samples (these are subject to overdispersion) as well as any available single-cell derived counts Y_i^{sc} that had also been recorded in Z. (Here we do not collapse by counting contributions over i, since such summarized counts would entail undue information loss; instead Y is a collection of vectors.) To emphasize notational distinctions, X, Y, and Z refer to random elements in our actual experiment, taking possible values x, y, and z. As is sometimes done in p-value discussions, we introduce a separate notation for data obtained from a hypothetical repeat of the experiment: let X^{rep} and Z^{rep} denote hypothetical repeated draws of the random vectors X and Z. In these terms, we originally considered

$$p_{\text{cp}}^*(x,y) = P\left\{ h(X^{\text{rep}}, Z^{\text{rep}}) \leq h(x,Z) \mid C, Y = y \right\} \tag{2}$$

where the event $C = \{X^{\text{rep}} + Z^{\text{rep}} = x + Z\}$, interpreting the sums in C as coordinate-wise additions. To investigate this probability, write the key event as

$$B = \{\, h(X^{\text{rep}}, Z^{\text{rep}}) \leq h(x, Z) \,\}$$

and note

$$\begin{aligned} p^*_{\text{cp}}(x, y) &= P(B|C, Y = y) \\ &= E\{\, P(B|C, Z, \theta, Y = y) \,|\, C, Y = y\} \end{aligned} \tag{3}$$

by iterated expectations. Here θ is the vector of unknown probabilities over types (its randomness/uncertainty comes from the Dirichlet prior distribution) and Z is the aggregated vector of true type counts. The inner conditional probability, at any realization θ and $Z = z$, is

$$P\{\, h(X^{\text{rep}}, Z^{\text{rep}}) \leq h(x, z) \,|\, C, \theta, Z = z, Y = y\}. \tag{4}$$

By sampling, X^{rep}, Z^{rep}, and Y are conditionally independent given θ, and so the event $Y = y$ may be erased from the conditioning event in (4). Further, the conditioning on C converts the conditionally independent multinomial vectors X^{rep} and Z^{rep} into a generalized hypergeometric table, and thus allows us to erase θ from the same conditioning event. By further simplification, (4) becomes

$$P\{\, h(X^{\text{rep}}, Z^{\text{rep}}) \leq h(x, z) \,|\, X^{\text{rep}} + Z^{\text{rep}} = x + z\}. \tag{5}$$

This is precisely the Fisher-exact-test p-value that one would compute from x and z, say $p_{\text{Fisher}}(x, z)$, and so we have established that the p-value defined in (2) satisfies:

$$p^*_{\text{cp}}(x, y) = E\{p_{\text{Fisher}}(x, Z)|\, C, Y = y\}\,.$$

Recognizing that C no longer affects this expression, we have finally

$$p^*_{\text{cp}}(x, y) = E\{p_{\text{Fisher}}(x, Z)|\, Y = y\}\,. \tag{6}$$

as suggested in the opening of this section.

For further analysis we consider a somewhat simpler data structure. Specifically, suppose that at the level of resolution of interest, there are $K \geq 2$ distinct types of T-cell receptors, and a single vector $X = (X_1, X_2, \ldots, X_K)^T$ counts occurrances of these types from a set of size $m = \sum_{k=1}^K X_j$ receptors sequenced from WT cells. Similarly, a single vector $Y = (Y_1, Y_2, \ldots, Y_K)^T$ records $n = \sum_{k=1}^K Y_k$ MT mass culture counts, and a vector $Z = (Z_1, Z_2, \ldots, Z_K)^T$ records the latent counts in MT cells immediately after selection in a mass culture experiment. As new notation, let $N = \sum_{k=1}^K Z_k$ count the total number of surviving MT cells. In contrast to the actual data structure, this one overlooks the breakdown of counts according to different patients, and it ignores any MT SC data.

Consider the $K \times 2$ contingency table with columns X and Z holding WT and MT counts, respectively. The null model asserts that X and Z are independent multinomial vectors from a common probability vector $\theta = (\theta_1, \theta_2, \ldots, \theta_K)^T$, given totals (row sums) m and N. We suppose that all types are possible; that is $\theta_k > 0$ for all k. It is well known that if m and N are reasonably large, then Fisher's exact p-value is equivalent to the p-value computed from Pearson's chi-square statistic (e.g., Bickel and Doksum, 1977, page 323). In this $K \times 2$ table, Pearson's statistic is

$$\sum_{k=1}^K \left[\frac{(X_k - m\hat{\theta}_k)^2}{m\hat{\theta}_k} + \frac{(Z_k - N\hat{\theta}_k)^2}{N\hat{\theta}_k} \right],$$

where $\hat{\theta}_k = (X_k + Z_k)/(m+N)$. This can be re-expressed as a quadratic form relative to the $K \times K$ diagonal matrix $D_{1/\hat{\theta}}$ holding $1/\hat{\theta}$ on its diagonal:

$$\frac{1}{\gamma_m + 1} (\tilde{X}_m - \sqrt{\gamma_m}\tilde{Z}_m)^T D_{1/\hat{\theta}} (\tilde{X}_m - \sqrt{\gamma_m}\tilde{Z}_m) \tag{7}$$

where $\gamma_m = m/N$ is a sampling ratio and $\tilde{X}$ and $\tilde{Z}$ are standardized versions of X and Z:

$$\tilde{X}_m = \sqrt{m}\left(\frac{X}{m} - \theta\right) \qquad \tilde{Z}_m = \sqrt{N}\left(\frac{Z}{N} - \theta\right). \tag{8}$$

Here we index by m because we envision asymptotics in which m and N agree in order of magnitude. We assume $\gamma_m \longrightarrow \gamma \in (0, \infty)$ as $m \longrightarrow \infty$. By the central limit theorem, the vectors $\tilde{X}_m$ and $\tilde{Z}_m$ in (8) converge in distribution to independent and identically distributed Gaussian vectors with mean zero and covariance matrix $D_\theta - \theta\theta^T$. Note that this covariance matrix is singular owing to the constraint $\sum_{k=1}^K \theta_k = 1$. Letting $\tilde{X}$ and $\tilde{Z}$ refer to the respective limits, the limiting chi-square statistic from (7) is

$$\chi^2(\tilde{X}, \tilde{Z}) = \frac{1}{\gamma + 1} (\tilde{X} - \sqrt{\gamma}\tilde{Z})^T D_{1/\theta} (\tilde{X} - \sqrt{\gamma}\tilde{Z}). \tag{9}$$

Since $\tilde{X} - \sqrt{\gamma}\tilde{Z}$ is Gaussian with mean zero and covariance matrix $(1+\gamma)\left(D_\theta - \theta\theta^T\right)$, it follows that $\chi^2(\tilde{X}, \tilde{Z})$ has a chi-square distribution with $K-1$ degrees of freedom, which is the trace of $B = D_{1/\theta}^{-1/2}(D_\theta - \theta\theta^T)D_{1/\theta}^{-1/2}$ (*e.g.*, Bishop, Fienberg, and Holland, 1975, page 473). Introducing an independent mean zero Gaussian vector U, with covariance matrix $D_\theta - \theta\theta^T$, the *would-be* Fisher p-value is

$$p_{\text{Fisher}}(\tilde{x}, \tilde{z}) = P\left[U^T D_{1/\theta} U \geq \chi^2(\tilde{x}, \tilde{z})\right] \tag{10}$$

when realized at $\tilde{X} = \tilde{x}$ and $\tilde{Z} = \tilde{z}$, since the quadratic form $U^T D_{1/\theta} U$ also has a chi-square distribution on $K-1$ degrees of freedom. Further, the realized value of the conditional predictive p-value is

$$p^*_{\text{cp}}(\tilde{x}, \tilde{y}) = E\left[p_{\text{Fisher}}(\tilde{x}, \tilde{Z}) \middle| \tilde{Y} = \tilde{y}\right] \tag{11}$$

for some limiting standardized version $\tilde{Y}$ of the MT mass culture counts Y. In the finite-sample case (equation 6), the expectation refers to the posterior predictive distribution that integrates both uncertainty in the parameters ϕ and θ as well as the missing counts Z. In the limiting case (11), the expectation is assumed to average only the latent $\tilde{Z}$. All parameter uncertainty is assumed to have vanished, as predicted by large-sample parametric Bayesian inference (e.g., Schervish, 1995, Section 7.4). Mimicking the finite-sample model structure, the standardized $\tilde{Y}$ satisfies:

$$\tilde{Y}\Big| \tilde{Z} = \tilde{z} \sim \text{Normal}\left[\tilde{z}, \xi^2(D_\theta - \theta\theta^T)\right]$$

where $\xi > 1$ reflects limiting extra-multinomial variation. This is precisely the limiting form implied by the Dirichlet-Multinomial observation model; ξ depends on the overdispersion parameter ϕ and the limit of the sampling ratio N/n (when the limiting sampling ratio is unity, $\xi^2 = 1 + 1/\phi$). Therefore, the conditional expectation in (11) is relative to the distribution

$$\tilde{Z}\Big| \tilde{Y} = \tilde{y} \sim \text{Normal}\left[\frac{\tilde{y}}{1+\xi^2}, \frac{\xi^2}{1+\xi^2}(D_\theta - \theta\theta^T)\right]. \tag{12}$$

This follows straightforward normal-theory regression, though accounting for the singularity of the covariance matrix.

There is benefit in taking a change-of-variables in (11) and (12). Introduce the random vector

$$V = \sqrt{\frac{1+\xi^2}{\xi^2}} \left(\tilde{Z} - \frac{\tilde{Y}}{1+\xi^2} \right).$$

Conditionally upon $\tilde{Y} = \tilde{y}$, V is a mean zero Gaussian with covariance matrix $D_\theta - \theta\theta^T$. Indeed this is also the marginal distribution of V. Various quantities can be re-written in terms of V. For example, with $a = \sqrt{\gamma\xi^2/(1+\xi^2)}$ the deviation vector inside (9) is

$$\tilde{X} - \sqrt{\gamma}\tilde{Z} = \left(\tilde{X} - \frac{\sqrt{\gamma}\tilde{Y}}{1+\xi^2} \right) - aV.$$

The quantity in parentheses, say $W = \tilde{X} - \frac{\sqrt{\gamma}\tilde{Y}}{1+\xi^2}$, holds information from the limiting observables, and V expresses limiting latent deviations. With these considerations, the limiting conditional predictive p-value (11) can be evaluated explicitly. Using $w = \left(\tilde{x} - \sqrt{\gamma}\tilde{y}/(1+\xi^2)\right)$ as a possible realization of W,

$$\begin{aligned} p^*_{\text{cp}}(\tilde{x}, \tilde{y}) &= P\left[U^T D_{1/\theta} U \geq \frac{1}{1+\gamma}(w - aV)^T D_{1/\theta}(w - aV) \right] \\ &= P\left[\frac{(V - w/a)^T D_{1/\theta}(V - w/a)}{U^T D_{1/\theta} U} \leq \frac{1+\gamma}{a^2} \right] \\ &= P\left[H \leq c + 1 \right] \end{aligned} \tag{13}$$

where H, being a ratio of independent chi-squared variables, has a non-central F distribution on $K-1$ and $K-1$ degrees of freedom, and with non-centrality parameter $\lambda = (w^T D_{1/\theta} w)/a^2$, and where $c + 1 = (1+\gamma)/a^2 = (1+\gamma)(1+\xi^2)/(\gamma\xi^2)$. This somewhat striking representation of the conditional predictive p-value enables us to study its limiting null probability distribution. Evidently the p-value depends on the data only through the quadratic form inside the non-centrality parameter λ. Randomness in the data is transduced through this non-centrality parameter, which becomes distributed as a scaler multiple of a (central on the null) chi-square random variable on $K-1$ degrees of freedom. Specifically, taking $S = (W^T D_{1/\theta} W)/[1+\gamma/(1+\xi^2)]$, we have established the following.

Theorem 1 *Let $G_\lambda(f)$ denote the cumulative distribution function of a non-central F distribution on $K-1$ and $K-1$ degrees of freedom, and with non-centrality parameter λ. With $c = \frac{1+\gamma}{\gamma}\frac{1+\xi^2}{\xi^2} - 1$, we have in the asymptotic null case that the conditional predictive p-value $p^*_{\text{cp}}(\tilde{X}, \tilde{Y})$ is equal in distribution to $G_{cS}(1+c)$, where $S \sim \chi^2_{K-1}$.*

The transformation $S \longrightarrow G_{cS}(1+c)$ is monotone decreasing, owing to properties of the non-central F distribution. It follows, therefore, that if s_α is a chi-square quantile such that $P(S > s_\alpha) = \alpha$ for $\alpha \in (0,1)$, then $G_{cS}(1+c) < G_{cs_\alpha}(1+c)$ when $S > s_\alpha$, and thus $P\left[G_{cS}(1+c) \leq G_{cs_\alpha}(1+c)\right] = \alpha$. That is, $G_{cs_\alpha}(1+c)$ is the α-quantile of the distribution of the limiting $p^*_{\text{cp}}(\tilde{X}, \tilde{Y})$. Introducing $F(p) = P[p^*_{\text{cp}}(\tilde{X}, \tilde{Y}) \leq p]$ as the limiting distribution function of the conditional predictive p-value, and letting F^{-1} be its quantile function, we have established a corollary to Theorem 1: namely, $F^{-1}(\alpha) = G_{cs_\alpha}(1+c)$. Figure S4 shows a few examples of the distribution function $F(p)$, which

is readily obtained by numerical inversion. Conservativeness of the limiting p-value is evident in these examples, since $F(p) < p$ for most p. We have not found a general proof, except when considering a neighborhood of the origin and invoking the inverse function theorem. On one hand, $\frac{d}{d\alpha}F^{-1}(\alpha) = \frac{1}{f[F^{-1}(\alpha)]}$, where f is the density function of $p_{\text{cp}}(\tilde{X}, \tilde{Y})$. Using the Poisson-mixture representation of G_λ, direct differentiation also gives

$$\begin{aligned}
\frac{d}{d\alpha}F^{-1}(\alpha) &= \frac{d}{d\alpha}G_{cs_\alpha}(1+c) \\
&= \frac{d}{d\alpha}\left[\exp\left\{\frac{-cs_\alpha}{2}\right\}\sum_{j=0}^{\infty}\frac{q_j}{j!}\left(\frac{cs_\alpha}{2}\right)^j\right] \\
&= \left(\frac{ds_\alpha}{d\alpha}\right)\left(\frac{c}{2}\right)\exp\left\{\frac{-cs_\alpha}{2}\right\}\sum_{j=0}^{\infty}\frac{(q_{j+1}-q_j)}{j!}\left(\frac{cs_\alpha}{2}\right)^j.
\end{aligned}$$

Here q_j is the probability that a *central* F distribution on $K-1+2j$ and $K-1$ degrees of freedom is less than $(1+c)/[1+2j/(K-1)]$. The behavior of F^{-1} is determined by the first factor $\frac{ds_\alpha}{d\alpha}$, which diverges as $\alpha \to 0$. Coupled with the first finding about F^{-1} we conclude that the p-value's density $f(p)$ converges to 0 as $p \to 0$, which further implies the following.

Theorem 2 *The conditional predictive p-value is dominated by the uniform distribution, asymptotically, in the sense that*

$$F(p) = P\left[p^*_{\text{cp}}(\tilde{X}, \tilde{Y}) \le p\right] < p \quad \text{for all sufficiently small } p > 0.$$

On the surface it seems that p^*_{cp} will work fine. It has a non-degenerate asymptotic null distribution that gives conservative p-values, and knowing the limit could enable a computational adjustment to uniformity. However something quite unusual happens because the asymptotic argument above has assumed knowledge of the underlying parameter vector θ in the probability simplex. This ordinarily would not be a problem, since we have a consistent estimate $\hat{\theta}$ available from the MT data, and thus we would do the statistical thing and simply plug in $\hat{\theta}$ where necessary to get an empirical version of p^*_{cp}. It turns out that this plug in completely destroys the asymptotic distribution of the p-value, in contrast to most plug in efforts. The reason is that with a large sample size, any deviation between θ and $\hat{\theta}$ is amplified by the (now) hyper-sensitive Fisher p-value on the large imputed data set. Computational experiments (not shown) bear this out. Fortunately, the proposed p-value p_{cp}, which does not average Fisher p-values, is not subject to this sampling defect.

Additional references

Albertini MR, Macklin MD, Zuleger CL, Newton MA, Judice SA, Albertini RJ. Clonal expansions of 6-thioguanine resistant T lymphocytes in the blood and tumor of melanoma patients. Environ Mol Mutagen 2008;49:676-87.

Bickel, P.J. and Doksum, K.A. (1977). *Mathematical Statistics: Basic ideas and selected topics*, Englewood Cliffs, New Jersey: Prentice Hall.

O'Neill JP, McGinniss MJ, Berman JK, Sullivan LM, Nicklas JA, Albertini RJ. Refinement of a T-lymphocyte cloning assay to quantify the in vivo thioguanine-resistant mutant frequency in humans. Mutagenesis 1987;2:87-94.

R Development Core Team (2011). *R: A language and environment for statistical computing.* Vienna, Austria: R Foundation for Statistical Computing, ISBN 3-900051-07-0. http://www.R-project.org/.

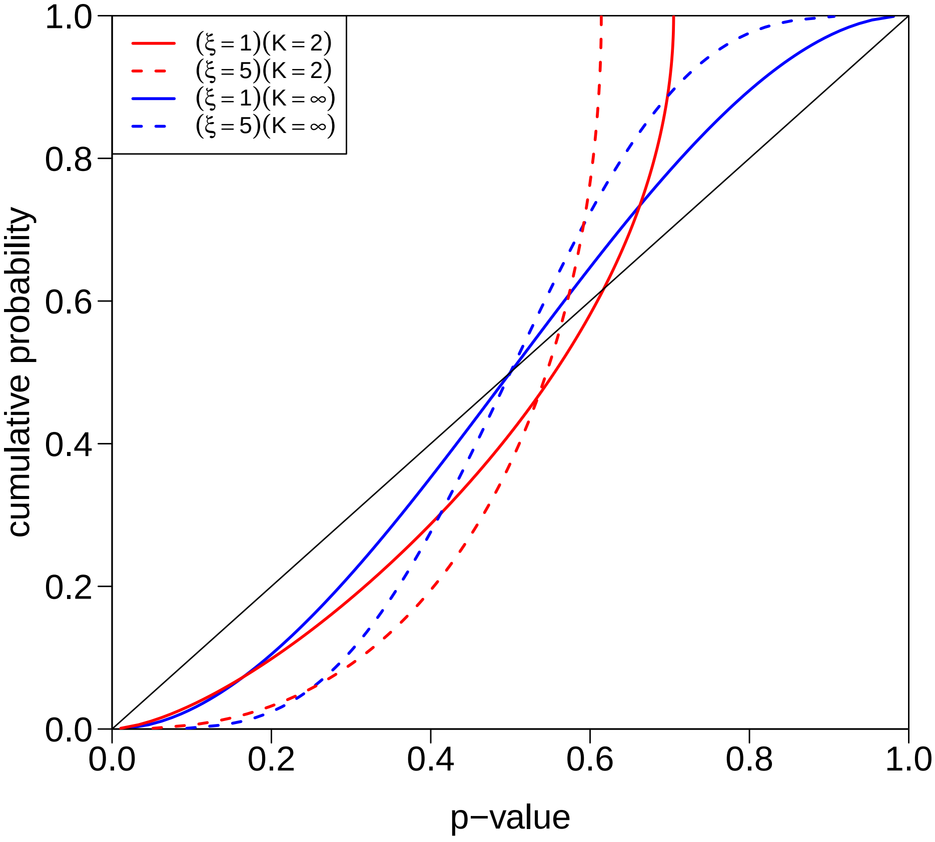

Figure S4: Limiting distribution functions of the (original) conditional predictive p-value p^*. Shown are four versions of $F(p)$, the limiting null c.d.f.. From Theorem 6.1, $F^{-1}(\alpha) = G_{cs_\alpha}(1 + c)$ for s_α an upper quantile of the chi-square distribution on $K - 1$ degrees of freedom, G_λ the c.d.f. of a non-central F distribution on $K - 1$ and $K - 1$ degrees of freedom, with non-centrality parameter λ, and a constant $c > 0$ that depends on the extent of overdispersion, as measured by ξ. In all cases investigated, the c.d.f. is dominated by the uniform distribution for small p, as predicted by Theorem 2. The greater the overdispersion the larger the deviation from uniformity. The number of distinct T-cell types K has a modest effect on the distribution. An explicit formula for $F(p)$ is available in the $K \to \infty$ case.
